# Quantification, description and international comparison of antimicrobial use on Irish pig farms

**DOI:** 10.1186/s40813-020-00166-y

**Published:** 2020-10-12

**Authors:** Lorcan O’Neill, Maria Rodrigues da Costa, Finola C. Leonard, James Gibbons, Julia Adriana Calderón Díaz, Gerard McCutcheon, Edgar García Manzanilla

**Affiliations:** 1 Pig Development Department, Teagasc, The Irish Food and Agriculture Authority, Moorepark, Fermoy, Co Cork Ireland; 2 School of Veterinary Medicine, University College Dublin, Belfield, Dublin 4, Ireland; 3 Irish Equine Centre, Johnstown, Co Kildare Ireland; 4 Pig Development Department, Teagasc, The Irish Food and Agriculture Authority, Oakpark, Carlow, Co Carlow Ireland

**Keywords:** Antimicrobial use, Pigs, Ireland

## Abstract

**Background:**

There is concern that the use of antimicrobials in livestock production has a role in the emergence and dissemination of antimicrobial resistance in animals and humans. Consequently, there are increasing efforts to reduce antimicrobial use (AMU) in agriculture. As the largest consumer of veterinary antimicrobials in several countries, the pig sector is a particular focus of these efforts. Data on AMU in pig production in Ireland are lacking. This study aimed to quantify AMU on Irish pig farms, to identify the major patterns of use employed and to compare the results obtained to those from other published reports and studies.

**Results:**

Antimicrobial use data for 2016 was collected from 67 Irish pig farms which represented c. 35% of national production. The combined sample population consumed 14.5 t of antimicrobial by weight of active ingredient suggesting that the pig sector accounted for approximately 40% of veterinary AMU in Ireland in 2016. At farm level, median AMU measured in milligram per population correction unit (mg/PCU) was 93.9 (range: 1.0–1196.0). When measured in terms of treatment incidence (TI200), median AMU was 15.4 (range: 0.2–169.2). Oral treatments accounted for 97.5% of all AMU by weight of active ingredient and were primarily administered via medicated feed to pigs in the post weaning stages of production. AMU in Irish pig production in 2016 was higher than results obtained from the national reports of Sweden, Denmark, the Netherlands and France but lower than the United Kingdom.

**Conclusions:**

Pig production in Ireland is an important consumer of veterinary antimicrobials. The quantities and patterns of AMU on Irish pig farms are comparable to pig production in other European countries but higher than some countries with more advanced AMU reduction strategies. This AMU is characterised by a high proportion of prophylactic use and is primarily administered to pigs post weaning via medicated feed. Further studies to better understand the reasons for AMU on Irish pig farms and strategies to improve health among weaner pigs will be of benefit in the effort to reduce AMU.

## Background

In the global effort to combat antimicrobial resistance a ‘One Health’ approach, encompassing human and animal health as well as the environment, has been proposed [1, 2]. In common with other countries, Ireland has implemented its own One Health action plan, iNAP (Ireland’s National Action Plan against Antimicrobial Resistance) [3]. Efforts to monitor and ultimately reduce antimicrobial use (AMU) in livestock production are important components of such plans [4]. These efforts are necessary as the use of antimicrobials in livestock production has been linked to the emergence and dissemination of antimicrobial resistant bacteria in animals and humans [5–7].

Pig production is the highest consumer of veterinary antimicrobials in many countries [8–10]. Until recently, only a limited number of countries, such as Denmark [8] and the Netherlands [9], monitored AMU stratified by species. Currently, however, many countries have followed the example of Denmark and the Netherlands and have developed, or are developing, their own AMU databases [11]. Data from some of these schemes were published recently, for example, from Germany [12] and Belgium [10]. Elsewhere, data on AMU in pig production derives from a limited number of cross-sectional studies in countries such as Canada [13, 14], Austria [15], France [16, 17], Spain [18, 19] and Belgium [20] as well as two pan European studies [21, 22]. Several of these studies show that the majority of antimicrobials are administered orally, as group treatments, are frequently applied for prophylactic (to prevent disease) or metaphylactic purposes (to treat a group containing some diseased animals) and primarily administered to pigs post weaning.

The pig population in Ireland comprises approximately 140–150,000 breeding animals and 1.5–1.6 million fattening pigs and is the third largest livestock sector after dairy and beef [23, 24]. To date, data on the use of antimicrobials in Irish pig production are lacking but is reputed to be high [25].

The objectives of this study were 1) to quantify antimicrobial use on Irish pig farms, 2) to determine the major patterns of use employed, and 3) to compare the results to those from other published reports and studies.

## Methods

### Farm selection

A cross-sectional study was conducted on a convenience sample of 67 Irish pig farms to investigate antimicrobial usage. The participating farms were clients of the Teagasc (The Agriculture and Food Development Authority) farm advisory service^1^ which is available to all Irish pig farms. In 2017, the Teagasc farm advisory service included 107 pig farms, representing over 77,000 sows (c. 50% of national herd); all farms were invited to participate in the study and 67 agreed to co-operate. All farms operated a farrow-to-finish system. Farrow-to-finish enterprises account for virtually all of pig production in Ireland [26].

### Data collection

Farms were visited between September 2017 and October 2018 to collect detailed antimicrobial use data for the 2016 calendar year. The farmers were asked to provide prescription and or invoice data in order to determine the amounts of antimicrobials used. Farmers were asked to indicate which stages of production each antimicrobial preparation was used in and whether any prophylactic or metaphylactic use occurred during the year. Population, feed consumption, performance and production data were obtained from the Teagasc e-Profit Monitor (ePM) database to which farmers submit their data quarterly. Farmers not using the ePM (*n* = 8) were asked to provide the relevant data directly.

### Quantification of antimicrobial use

The amounts of active ingredient in each antimicrobial product were determined according to the protocols outlined by the European Medicines Agency (EMA) for its European Surveillance of Veterinary Antimicrobial Consumption (ESVAC) project [27]. Conversion factors for prodrugs (such as procaine benzylpenicillin) and international units (I.U.) were also obtained from the ESVAC protocol.

#### Medicated feed

Antimicrobial oral premixes are remedies specifically intended for use in medicated feed [28] and thus are distinct from oral remedies intended for use in water or those added to feed as a ‘top dressing.’ While medicated feeds may include other types of medication (for example, anthelmintics, anti-inflammatories or zinc oxide), in this article, the term ‘medicated feed’ will refer to feeds or diets including antimicrobial oral premixes. Specific diets are fed during each stage of pig production and any of these diets may be medicated with antimicrobials. The stages of production on Irish pig farms are summarised as follows: piglets are generally weaned at around 28 days, the weaned piglets remain in the weaner stage, which, on Irish farms is typically split into first and second stages, for up to 9 weeks and, thereafter, the pigs stay in the finisher stage until slaughter, at around 24 weeks of age. A creep or pre-starter diet is provided to piglets in the farrowing house; after weaning, the pigs are typically fed a starter diet for 7–14 days, followed by a link diet for another 7–14 days and then a weaner diet for the remainder of the weaner stage; finally, finisher pigs are fed a finisher diet. Sows are fed specific diets depending on whether they are in gestation or lactation. The amount of medicated diet used in each category for each antimicrobial included was determined by 1) consulting data submitted to the ePM, 2) invoice records, or 3) data provided directly by the farmer. To calculate the amounts of antimicrobial administered in medicated feed the following formula was used:

$$ \mathrm{weight}\ \mathrm{of}\ \mathrm{medicated}\ \mathrm{feed}\ \left(\mathrm{kg}\right)\times \mathrm{inclusion}\ \mathrm{rate}\ \mathrm{of}\ \mathrm{active}\ \mathrm{ingredient}\ \left(\mathrm{mg}/\mathrm{kg}\right) $$

The inclusion rate was expressed in terms of mg of antimicrobial per kg of medicated feed (e.g. chlortetracycline 300 mg/kg). This calculation was performed for every combination of diet and antimicrobial inclusion rate.

#### Other oral remedies and parenteral preparations

Complete prescription records for other oral remedies and parenteral preparations were not available for five farms. In these cases, missing values for each antimicrobial product used on the farm were imputed based on either the estimate of use provided by the farmer or the median value of use on the other farms using the same product. To calculate the amounts of antimicrobials administered by other routes of administration the following formula was used:

$$ \mathrm{number}\ \mathrm{of}\ \mathrm{pack}\mathrm{s}\times \mathrm{pack}\ \mathrm{size}\ \left(\mathrm{g}\ \mathrm{or}\ \mathrm{ml}\right)\times \mathrm{strength}\ \left(\mathrm{mg}/\mathrm{ml}\ \mathrm{or}\ \mathrm{mg}/\mathrm{kg}\right) $$

This calculation was performed for each preparation of each active ingredient.

### Treatable kilograms

In order to adjust for differences in potency between the various active ingredients, the amounts used of each antimicrobial were adjusted to ‘treatable kilograms’ (TK) based on the Defined Daily Dose for the given active ingredient (DDD_vet_) as defined by ESVAC [29]. ‘Treatable kilograms’ represents the number of kilograms of pig which can be treated with the given amount of antimicrobial if the Defined Daily Dose is used. For example, 20 mg of marbofloxacin (DDD_vet_ = 2 mg/kg) can treat 10 kg of pig. This calculation is based on the definition outlined by the Netherlands Veterinary Medicines Institute [9]:

$$ \mathrm{treatable}\ \mathrm{kilograms}\ \left({\mathrm{TK}}_{\mathrm{DDD}\mathrm{vet}}\right)=\frac{\mathrm{amount}\ \mathrm{of}\ \mathrm{antimicrobial}\ \mathrm{used}\ \left(\mathrm{mg}\right)}{{\mathrm{DDD}}_{\mathrm{vet}}\left(\mathrm{mg}/\mathrm{kg}\right)} $$

Two antimicrobials, tulathromycin and tildipirosin, do not have an assigned DDD_vet_; the defined daily animal doses (DDDA) and long acting factors defined by Postma et al. were used [30].

### Indicators of antimicrobial use at farm level

The milligram per population correction unit (mg/PCU) was developed by the EMA and is the indicator of antimicrobial consumption used in the ESVAC reports on sales of veterinary antimicrobials in the European Union (EU) and European Economic Area (EEA) [31]. The mg/PCU uses the weight of active ingredient as the numerator while the population correction unit (PCU) is used as the denominator. The PCU assigns a standardised weight to each species and to sub-categories where applicable [31]. The calculation of mg/PCU for pigs is further described in Additional file 1.

Treatment incidence (TI) represents the percentage of pigs in a stage of production treated with a dose of antimicrobial each day or, the percentage time of the period at risk for which a pig was treated. The TI indicator, as defined for pigs by Timmerman et al. [32] and adapted by Sarrazin et al. [22], was calculated as follows:

$$ {\mathrm{TI}}_{\mathrm{DDD}\mathrm{vet}}=\frac{\mathrm{amount}\ \mathrm{o}\kern-0.1em \mathrm{f}\ \mathrm{antimicrobial}\ \mathrm{used}\ \left(\mathrm{mg}\right)}{{\mathrm{DDD}}_{\mathrm{vet}}\left(\mathrm{mg}/\mathrm{kg}\right)\times \mathrm{kg}\ \mathrm{o}\kern-0.1em \mathrm{f}\ \mathrm{animal}\ \mathrm{at}\ \mathrm{risk}\left(\mathrm{kg}\right)\times \mathrm{number}\ \mathrm{of}\ \mathrm{days}\ \mathrm{at}\ \mathrm{risk}}\times 100\ \mathrm{animal}\mathrm{s}\ \mathrm{at}\ \mathrm{risk} $$

The numbers of animals at risk and numbers of days at risk for each category were derived from ePM or farm data. For a detailed description of the TI calculation see Additional file 1. The TI_DDDvet_ was calculated for each antimicrobial per age category (piglet, weaner, finisher and sow). Finally, the TI for piglets, weaners and finishers were combined and recalculated as a standardised TI200 using the formula defined by Sjölund et al. [21]:

$$ \mathrm{TI}200=\frac{{\mathrm{TI}}_{\mathrm{piglet}}\times \mathrm{suckling}\ \mathrm{period}+{\mathrm{TI}}_{\mathrm{weaner}}\times \mathrm{weaner}\ \mathrm{period}+{\mathrm{TI}}_{\mathrm{finisher}}\times \mathrm{finishing}\ \mathrm{period}}{\mathrm{total}\ \mathrm{rearing}\ \mathrm{period}}\times \frac{200\left(\mathrm{standard}\ \mathrm{lifespan}\right)}{\mathrm{total}\ \mathrm{rearing}\ \mathrm{period}} $$

### Comparison to AMU in selected European countries

Data concerning the weight of antimicrobials used or sold in pig production during 2016 were extracted from the national reports of the following countries: Sweden [33], Netherlands [34], Denmark [35], and France [36]. The value obtained was divided by the corresponding PCU for pigs extracted from the ESVAC report for 2016 [37] to calculate consumption in mg/PCU. Antimicrobial use in pigs in the United Kingdom (UK) for 2016 was based on data collected by the e-medicine book for pigs (eMB pig) and was reported in mg/PCU [38].

To allow a further comparison with the national AMU consumption reports of Denmark and the Netherlands for 2016, the total AMU of the combined population of the sample farms was recalculated using their respective indicators. The DAPD, defined as the ‘proportion of population in treatment per day’, is the indicator used to report AMU at national level in Demark [8] and the DDDA_NAT_ (Defined Daily Dose Animal) is used in the national reports of the Netherlands [9]. For further details, see Additional file 1.

### Data processing

All data were entered into a Microsoft® Excel 365 spreadsheet. Calculations and descriptive statistical analysis were carried out using Microsoft® Excel and R version 3.4.2 [39]. Data visualisation was carried out using the R packages ggplot2 [40] and VennDiagram [41].

## Results

### Farm characteristics

The 67 farms included in the study had a median herd size of 528 sows (range 110–3000) and median production of 12,429 pigs for slaughter (range 2600–58,300) during 2016. The combined population of the 67 farms was approximately 48,000 sows and thus represented around 35% of the national herd in 2016 [42].

### Overview of antimicrobial consumption

The total estimate of antimicrobial use on the 67 study farms during 2016 by weight of active ingredient was 14.5 t, comprising a total of 19 different antimicrobial compounds. Table 1 summarises the patterns of antimicrobial use and shows the breakdown of use by route of administration for each antimicrobial class. The majority of antimicrobials, representing 97.5% of the weight of active ingredient and 93.9% of treatable kilograms (TK_DDDvet_), were administered orally and mainly in medicated feed. One farm did not use any oral treatments while all farms used injectable treatments. Tetracyclines, potentiated sulphonamides, macrolides and penicillins accounted for almost all antimicrobials consumed (98.2% of the weight of active ingredient; 94.3% of TK_DDDvet_). The use of tetracyclines in medicated feed had the highest impact on consumption, was observed on 64.1% of farms and accounted for more than half all AMU by weight of active ingredient (32.4% of TK_DDDvet_). Some patterns of use, while not impacting greatly on overall consumption, were observed on most farms. For example, all farms used injectable penicillins (amoxycillin or benzylpenicillin) and 83.6% of farms used injectable fluoroquinolones (enrofloxacin and marbofloxacin). Antimicrobial use was highest in weaner pigs, which accounted for 69.7% of AMU by weight of active ingredient and 63.2% of TK_DDDvet_. Finisher pigs accounted for 25.4% of AMU by weight of active ingredient and 30.6% of AMU by TK_DDDvet_; sows accounted for 4.1% of AMU by weight of active ingredient and 3.5% of AMU by TK_DDDvet_ and piglets accounted for 0.9% of AMU by weight of active ingredient and 2.7% of AMU by TK_DDDvet_ (see also Supplementary Table 1, Additional file 2). The indicators of AMU at farm level in mg/PCU and treatment incidence (TI200) are summarised in Table 2.

**Table 1:** Breakdown of antimicrobial use on 67 Irish farrow-to-finish pig farms during 2016 by route of administration and antimicrobial class. Percentages of active ingredient and TK_DDDvet_ refer to the percentage of overall consumption in terms of weight of active ingredient and number of treatable kilograms, respectively. Percentage of farms with use refers to the percentage of the 67 farms which used each combination of antimicrobial class and route of administration. Total consumption by weight of active ingredient was 14,505 kg; the corresponding number of treatable kilograms (TK_DDDvet_) was 775,128,079 kg. Mg/PCU in this table refers to consumption for the population of all 67 farms combined

		Overall (%)			Medicated feed (%)			Water (%)			Top dressing (%)			Oral dose (%)			Injectable (%)		
Antimicrobial class	mg/PCU	AI^a^	TK_DDDvet_^b^	Farms^c^	AI	TK_DDDvet_	Farms	AI	TK_DDDvet_	Farms	AI	TK_DDDvet_	Farms	AI	TK_DDDvet_	Farms	AI	TK_DDDvet_	Farms
Tetracyclines	90.38	55.81	34.02	85.07	53.72	32.43	64.18	0.84	0.51	7.46	1.07	0.65	23.88	–	–	–	0.17	0.43	41.79
Potentiated sulphonamides	40.85	25.22	33.86	46.27	25.15	33.74	34.33	–	–	–	0.04	0.05	2.99	0.01	0.01	5.97	0.03	0.07	13.43
Macrolides	15.09	9.32	16.02	65.67	5.99	10.10	37.31	3.28	5.11	13.43	–	–	–	–	–	–	0.04	0.80	34.33
Penicillins	12.69	7.84	10.39	100	3.68	4.57	50.75	2.48	2.73	32.84	0.01	0.01	2.99	–	–	–	1.68	3.09	100
Aminoglycosides	1.40	0.87	1.41	74.63	0.35	0.72	20.90	0.18	0.37	41.79	<0.01	<0.01	1.49	–	–	–	0.34	0.32	47.76
Amphenicols	0.54	0.33	0.62	19.40	0.09	0.17	5.97	0.21	0.39	5.97	–	–	–	–	–	–	0.03	0.06	10.45
Polymyxins	0.29	0.18	0.68	20.90	–	–	–	0.18	0.68	20.90	–	–	–	–	–	–	–	–	–
Aminocyclitols	0.24	0.15	0.67	53.73	0.10	0.53	13.43	0.02	0.12	10.45	–	–	–	0.02	0.01	31.34	0.01	0.01	19.40
Lincosamides	0.22	0.13	0.97	56.72	0.10	0.82	13.43	0.01	0.09	10.45	–	–	–	–	–	–	0.03	0.05	44.78
Fluoroquinolones	0.22	0.13	0.86	85.07	–	–	–	–	–	–	–	–	–	< 0.01	< 0.01	5.97	0.13	0.86	83.58
3rd & 4th gen. Cephalosporins	0.03	0.02	0.49	23.88	–	–	–	–	–	–	–	–	–	–	–	–	0.02	0.49	23.88
Pleuromutilins	< 0.01	< 0.01	< 0.01	1.49	–	–	–	–	–	–	–	–	–	–	–	–	<0.01	<0.01	1.49
TOTAL	**161.94**	**100**	**100**	**100**	**89.18**	**83.09**	**91.04**	**7.20**	**10.01**	**64.18**	**1.11**	**0.70**	**26.87**	**0.03**	**0.02**	**35.82**	**2.48**	**6.18**	**100**

^a^Active ingredient

^b^Treatable Kilograms (using the defined daily dose, DDD_vet_ [29])

^c^Farms with use

**Table 2:** Summary of antimicrobial use at farm level expressed in mg/PCU and in treatment incidence (TI) per age category (piglet, weaner, finisher and sow) and the standardised 200-day rearing period (TI200). Median values are shown with minima and maxima in brackets

	mg/PCU	TI200	TI_**piglet**_	TI_**weaner**_	TI_**finisher**_	TI_**sow**_
**Overall**	93.93 (1.01–1196.00)	15.37 (0.22–169.15)	6.32 (0.28–72.42)	34.36 (0.18–237.64)	0.70 (0.01–130.84)	0.19 (0.01–7.52)
**Medicated feed**	78.25 (0–1023.10)	12.87 (0–154.93)	1.29 (0–11.86)	32.54 (0–233.18)	0 (0–104.77)	0 (0–7.47)
**Water**	0.40 (0–150.79)	0.3 (0–33.12)	0.16 (0–69.74)	0.28 (0–42.78)	0 (0–25.94)	0 (0–1.78)
**Top dressing**	0.03 (0–29.15)	0 (0–1.39)	0 (0–0)	0 (0–1.53)	0 (0–1.28)	0 (0–2.25)
**Oral doses**	0 (0–0.72)	0 (0–0.24)	0 (0–1.19)	0 (0–0)	0 (0–0)	0 (0–0)
**Injectable**	3.91 (0.47–19.36)	1.17 (0.14–11.42)	2.43 (0.27–36.39)	0.58 (0.05–9.72)	0.32 (0.01–3.92)	0.10 (0.01–1.03)
**EMA category B antimicrobials** ^**a**^	0.18 (0–5.77)	0.24 (0–13.13)	0.39 (0–64.27)	0.16 (0–4.78)	0 (0–0.80)	0 (0–0.49)

^a^European Medicines Agency category B antimicrobials include polymyxins, fluoroquinolones and 3rd and 4th generation cephalosporins [43]

### Antimicrobial use in medicated feed

**Fig. 1:**
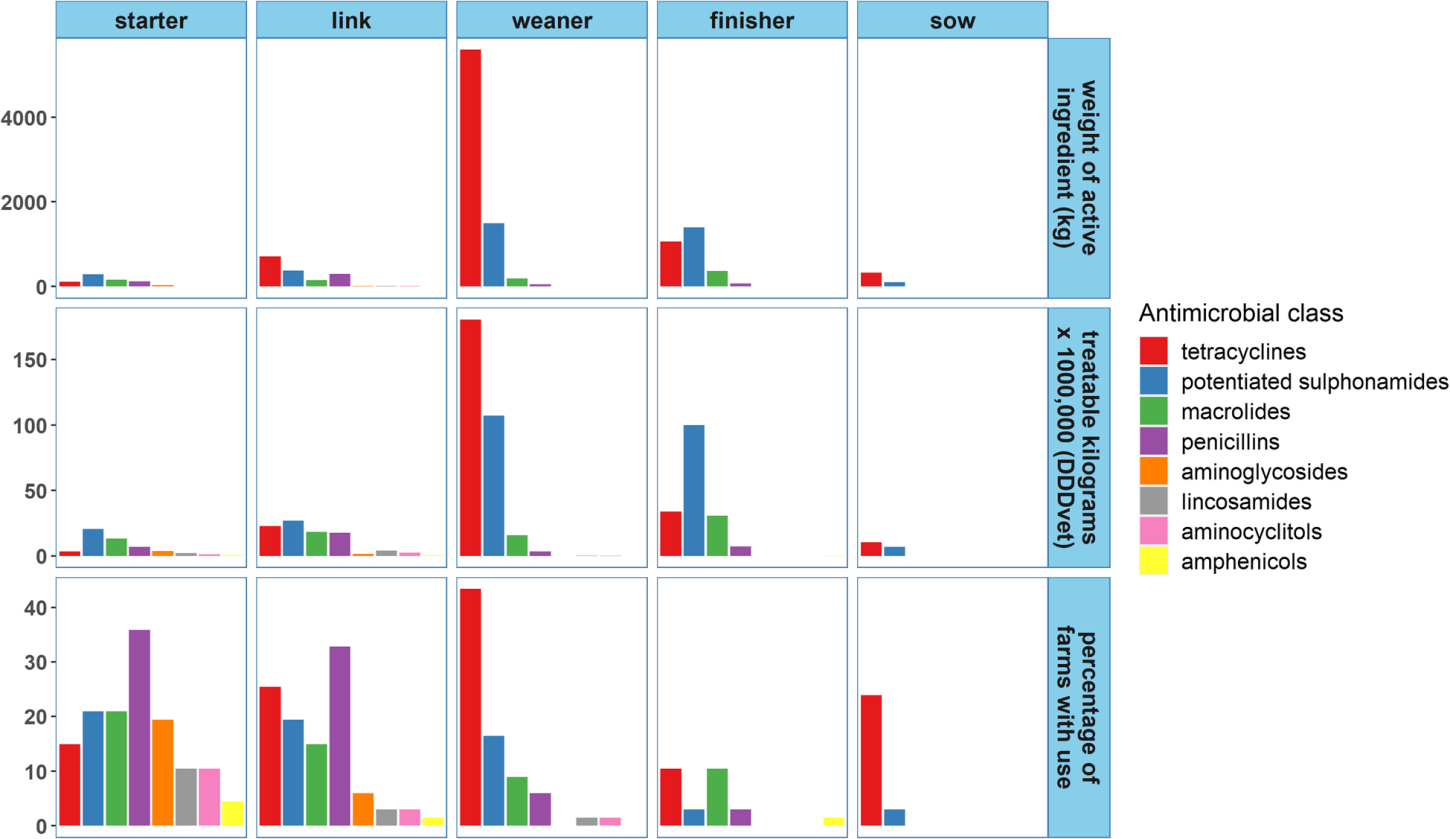
Consumption of the various classes of antimicrobials in medicated feed on 67 Irish pig farms, 2016. The data is stratified by weight of active ingredient, the number of treatable kilograms and the percentage of farms with use for each category of diet. Six farms did not use medicated feed during 2016 and some farms used more than one antimicrobial in a given diet during the year

**Fig. 2:**
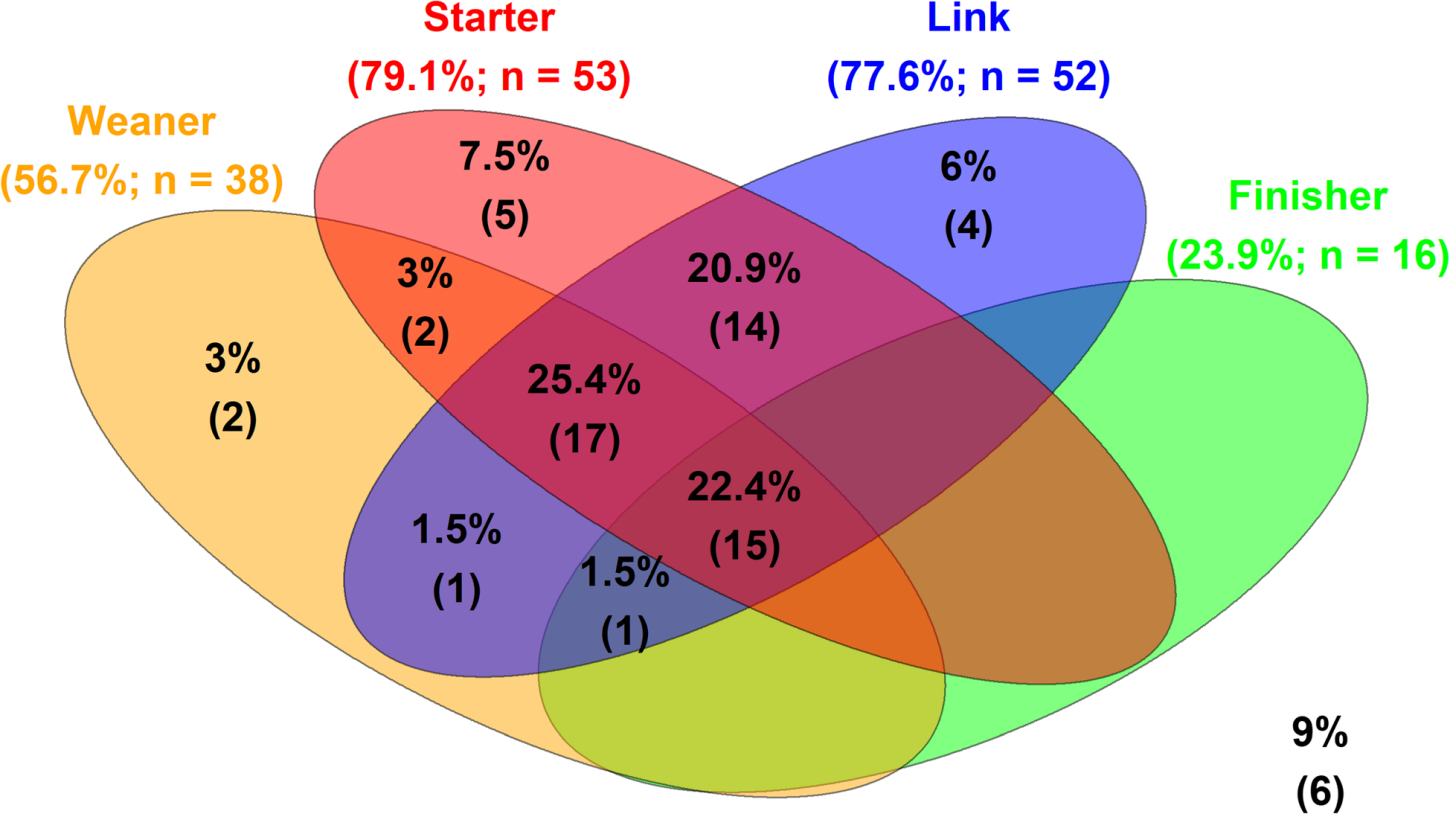
Venn diagram showing patterns of antimicrobial use in medicated feed for growing pigs on 67 farms, 2016. Each set contains the farms medicating the given diet category during 2016: starter, link, weaner and finisher. The percentage and number of farms medicating each diet category are shown in parentheses after the corresponding set title. The values within the sets show the percentage of farms (number in parentheses) with each pattern of use. For example, 6% of the study farms (*n* = 4) medicated the link diet only; 22.4% of the study farms (*n* = 15) medicated the starter, link, weaner and finisher diets. The values outside of the 4 sets indicate farms that did not use medicated diets

Figure 1 summarises AMU in medicated feed for each diet category. Sixty-one farms (91% of the sample) used medicated feeds during 2016 and this accounted for 89.1% of all AMU by weight of active ingredient (83.1% of TK_DDDvet_). The percentage of farms medicating each diet category and the patterns of use employed are illustrated in Fig. 2. The majority of medicated feed was provided to pigs in the post weaning stages of production and accounted for 66.3% of all AMU by weight of active ingredient (58.6% of TK_DDDvet_). Most farms (88.1% of sample) medicated at least the starter and/or link diets, meaning that most pigs in the study were treated with antimicrobials during the first 7–21 days post weaning. Thirty-five farms (52.2% of sample) also provided medicated creep diets to piglets in the farrowing house. Antimicrobial use in the starter and link diets accounted for 14.8% of weight of active ingredient and 19.0% of TK_DDDvet_. Regarding the active ingredients used, there was more variation in these diets compared to the other diet categories; tetracyclines, potentiated sulphonamides, macrolides, penicillins and aminoglycosides were all commonly used. Medicated weaner diets, mainly with tetracyclines or potentiated sulphonamides, were used on 38 farms (57.7% of sample). They generally followed medication in the starter and/or link diets (see Fig. 2) and accounted for 50.5% of total AMU by weight of active ingredient (39.6% of TK_DDDvet_). Antimicrobial use in medicated feed in the weaner stages was generally for prophylactic purposes; farms applied the same protocol to every batch produced during the year. In the finisher stage, medicated feed was used on 16 farms (23.9% of sample) and contributed to 20% of overall use by weight of active ingredient (22.3% of TK_DDDvet_). Tetracyclines and macrolides were the most commonly used classes in finisher diets, however, heavy use of sulfadiazine and trimethoprim on one farm meant it was the antimicrobial with the highest consumption in this category. Routine prophylaxis was less common and practised by six farms. In addition, 25.4% of farms provided medicated feed to sows, typically once or twice per year for a period of 7–10 days. The antimicrobials used in medicated feed varied within the farm, between diets and over time. Half of all farms used 3 or more antimicrobials (see Supplementary Figure 1, Additional file 2). This reflects the fact that some farms used combinations of antimicrobials, either combination products or custom formulations; that some farms changed treatment regime during the year and, that the antimicrobials included often varied from one diet to the next. Twenty-three of the 46 farms routinely medicating both the starter and link diets used the same treatment regime in both diets; of those farms that also medicated the weaner diet, all but one farm used a different antimicrobial.

### Antimicrobial use in other routes of administration

**Fig. 3:**
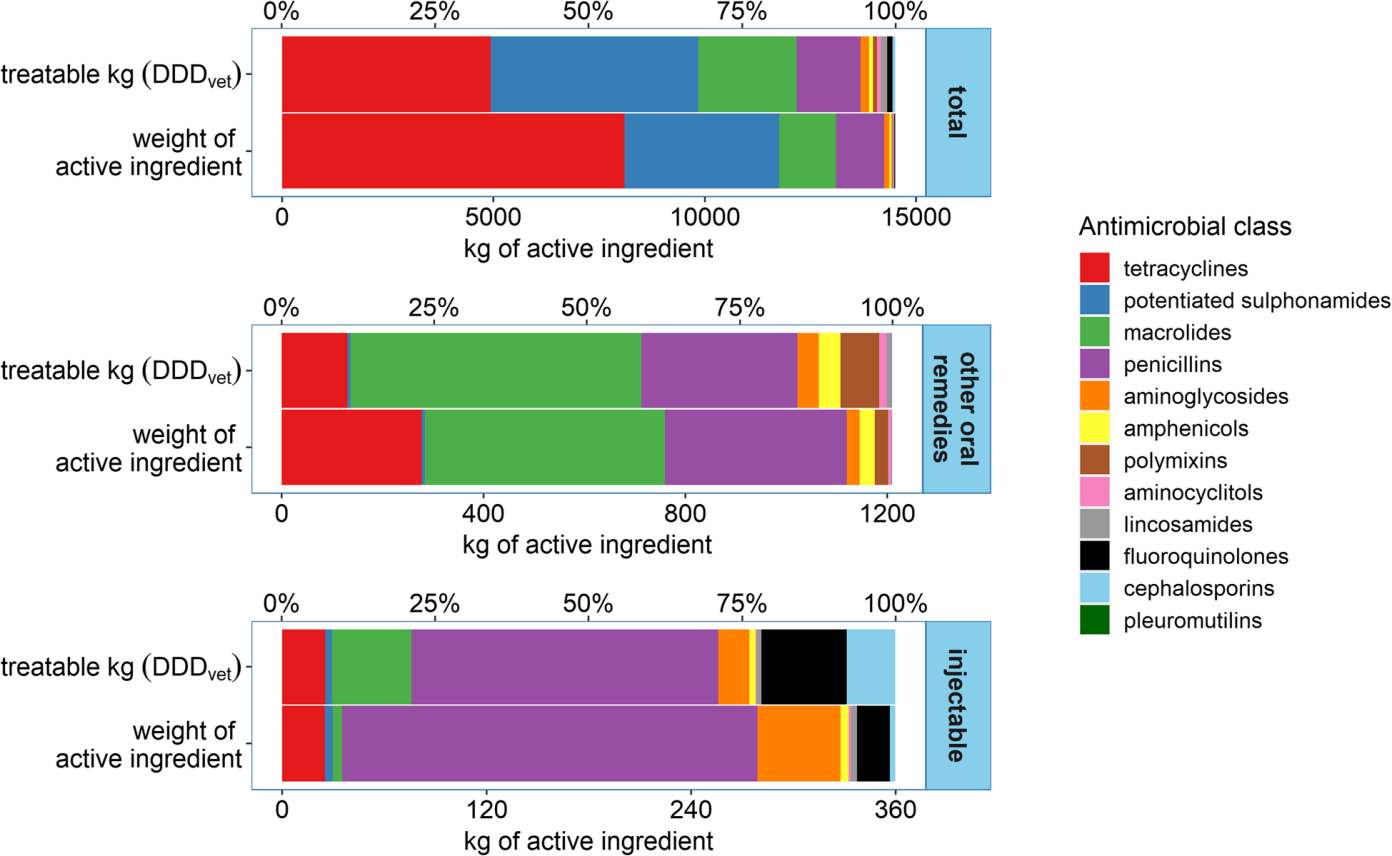
Consumption of oral remedies other than premix and parenteral antimicrobials expressed in weight of active substance and treatable kilograms (TK_DDDvet_) from 67 Irish pig farms, 2016. Other oral remedies include oral remedies for inclusion in water, oral powders for inclusion in feed as a ‘top dressing’ and, oral doses

The use of antimicrobials in injectables and oral remedies (other than premix) in weight of active ingredient and treatable kilograms is illustrated in Fig. 3. Forty-three farms (64.2% of sample) administered antimicrobials in water. This route of administration accounted for 7.2% of consumption by weight of active ingredient (10% of TK_DDDvet_). Macrolides and penicillins were the antimicrobial classes with the highest consumption (see Table 1). The most frequent practice was the administration of the aminoglycoside antimicrobial, apramycin, which was typically provided in drinkers in the farrowing house or post weaning to treat gastroenteritis and was used on 41.8% of farms. Top dressing, mainly with chlortetracycline, was typically administered to older weaners, finishers or sows, accounted for 1.11% of AMU by weight (0.7% of TK_DDDvet_) and was used on 16 farms (23.9% of sample). Oral dosing was recorded on 21 farms (31.3% of sample) and accounted 0.03% of AMU by weight of active ingredient and 0.02% of TK_DDDvet_.

Injectable antimicrobial remedies represented the third most important route of administration. Penicillins were the most used injectable antimicrobial class; all farms used amoxicillin and/or benzylpenicillin (+/− streptomycin). When measured by weight of active ingredient injectables accounted for 2.5% of all AMU and 6.2% of TK_DDDvet_.

### Highest priority critically important antimicrobials

The highest priority critically important antimicrobials (HP CIA), as defined by the World Health Organisation (WHO) [43], used on Irish pig farms included: macrolides; fluoroquinolones (enrofloxacin, marbofloxacin); third generation cephalosporins (ceftiofur); and polymyxins (colistin). The oral macrolides, mainly tylosin but also tilmicosin, represented 9.3% of all AMU by weight of active ingredient and 15.2% of TK_DDDvet_. The long acting injectable macrolides, tulathromycin and tildipirosin, were not widely used but nevertheless accounted for 12.5% of injectable TK_DDDvet_. The EMA classifies the fluoroquinolones, polymyxins and 3rd and 4th generation cephalosporins as category B antimicrobials (‘restrict’) and macrolides as category C (‘caution’) and thus differs from the WHO classification by ranking macrolides lower than the other HP CIAs [44]. In terms of overall use, category B antimicrobials represented 0.33% of the weight of active ingredient used; however, when measured in TK_DDDvet_ they accounted for 2% of consumption (see Table 1). Seven farms (10.4% of sample) did not use any category B antimicrobials during 2016 while most farms (85.1% of sample) used fluoroquinolones. The use of category B antimicrobials at farm level is summarised in Table 2. These were mainly administered to suckling piglets and weaned piglets where the highest treatment incidences were observed. In contrast, most farms did not administer category B antimicrobials to finisher pigs or to sows; median TIs in both of these age groups was zero.

### Prophylactic use

In addition to prophylactic AMU in medicated feed, farmers practised other methods of prophylactic treatment. Piglets were injected at birth, processing (iron injection, clipping teeth and tail docking; typically performed during the first week of life) or at weaning on 35 farms (52% of sample). Amoxicillin was the most commonly used drug (*n* = 16; 23.8% of sample) but five other classes of antimicrobial were utilised, notably: ceftiofur on six farms and long acting macrolides on five farms. In the weaner stage, other oral remedies, mainly amoxicillin (*n* = 4), chlortetracycline (*n* = 2) and tylosin (*n* = 1) were used as prophylaxis on seven farms (10.4% of sample). In finisher pigs, similar practices were observed on three farms: amoxicillin (*n* = 1); tylosin (*n* = 2). Sows received prophylactic treatments on 11 farms (16.4% of sample), most frequently in the form of an antimicrobial injection at either farrowing or weaning (eight of 11 farms). Oral chlortetracycline was administered to sows on four of the 11 farms. These practices accounted for a significant proportion of their respective routes of administration, for example prophylactic use of water soluble antimicrobials accounted for 78.8% of use in this route of administration by weight of active ingredient (for further details see Supplementary Table 2, Additional file 2).

### Comparison of AMU in Irish pig production to other countries

**Fig. 4:**
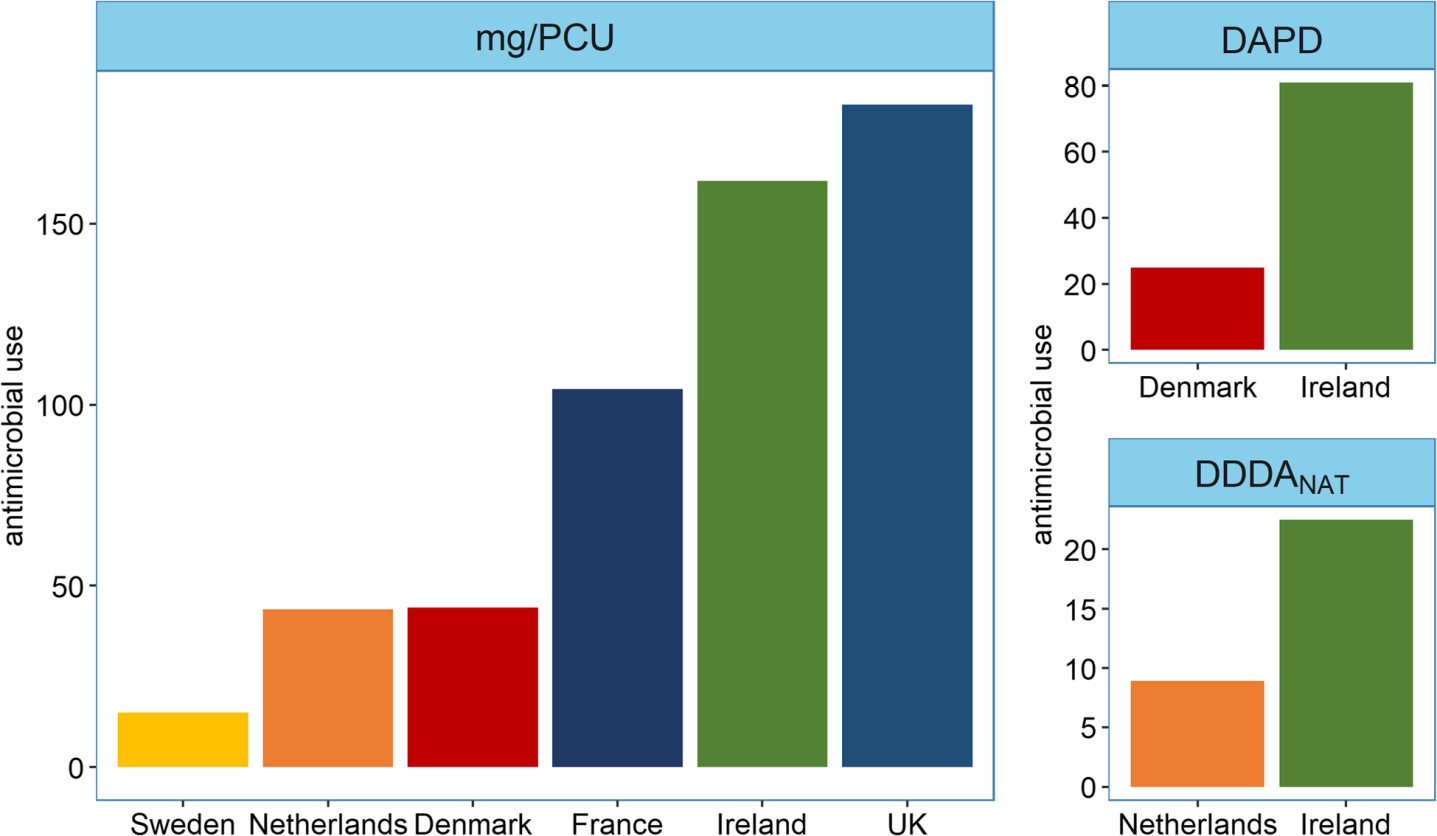
Comparison of antimicrobial use between the 67 Irish pig farms and 5 European countries in 2016. The large panel shows the comparison of antimicrobial use (AMU) between the Irish sample population and five European countries expressed in mg/PCU [31]. The smaller panels show the comparison of AMU between the Irish sample population and Denmark expressed in DAPD (proportion of animal population in treatment per day) [8] and between the Irish sample population and the Netherlands expressed in DDDA_NAT_ (defined daily dose animal) [9]

Figure 4 illustrates the comparison between AMU on the Irish sample farms and AMU as derived from the reports of Sweden, Denmark, the Netherlands, France and the UK in 2016. Antimicrobial use for the combined sample population of 67 Irish pig farms was 161.9 mg/PCU and was the second highest of the 6 countries behind the UK. Figure 4 also summarises the comparison of AMU between the Irish sample population and the Danish and Dutch pig production sectors using their respective national indicators. When measured in mg/PCU, AMU on the Irish pig farms was approximately 3.7 times higher than consumption in both Denmark and the Netherlands. However, when measured in their respective national indicators, DAPD and DDDA_NAT_, the relative differences were reduced. For the Irish study sample population, the DAPD was 3.2 times higher than that for Danish pig production while the DDDA_NAT_ was 2.5 times higher than Dutch pig production.

## Discussion

This study presents the first detailed data on antimicrobial use in the Irish pig industry. The study sample represented approximately 35% of the national herd and while it cannot be assumed to be representative of the whole of Irish pig production, the results obtained provide useful insights into antimicrobial use in the industry. Whether measured by weight of active ingredient or in defined daily doses (TK_DDDvet_) almost all antimicrobials were administered orally and primarily to pigs in the weaner stage of production. This is in agreement with several European studies [17–22, 45] and national reports [8, 9]. Approximately 103 t of veterinary antimicrobials were sold in Ireland during 2016, based on sales data submitted by Marketing Authorisation Holders to the Health Products Regulatory Authority [46]. Extrapolation of the results from this study suggests that pig production accounts for approximately 40% of veterinary AMU in Ireland. While this estimate must be interpreted with caution, the data from this study suggest that pigs consume all the oral premix antimicrobials (which accounted for one third of all sales in 2016) but are not significant consumers of the other oral remedies or injectable antimicrobials (which accounted for 33 and 27% of all sales respectively) [46].

Irish pig farms were also similar to European farms in that so called ‘older classes’ of antimicrobials such as tetracyclines, potentiated sulphonamides, penicillins, and macrolides were the most used. Macrolides, the third most used class overall, are classified as HP CIA by the WHO but category C (‘caution’) by the EMA. The difference in ranking between the EMA and WHO results from the EMA’s assessment of the importance of this class to veterinary medicine and the fact that for some conditions macrolides represent the only suitable treatment [44]. Colistin, classified as a HP CIA by the WHO [42] and category B (‘restrict’) by the EMA [44] and widely used in European pig production [21, 22] was not widely used on Irish farms. Use of cephalosporins and fluoroquinolones (both HP CIA and category B) was also relatively low; however, in countries such as the Netherlands [9] and Denmark [8] where there are restrictions, their use in pig production is negligible or zero. Furthermore, fluoroquinolones along with cephalosporins, constituted 21.8% of injectable AMU when measured in TK_DDDvet_. Since piglet was the age group with the highest exposure to injectable antimicrobials, this implies large proportions of the pig population may receive treatment with these drugs at some point in their life cycle. The widespread use of fluoroquinolones, in particular, is concerning as there is evidence of increasing fluoroquinolone resistance amongst *Escherichia coli* isolates of porcine origin in Ireland. Data from the European Food Safety Authority (EFSA) monitoring program showed that resistance to ciprofloxacin increased from 2% in 2015 to 7% in 2017 [47, 48].

In this study, 95.6% of all AMU by weight of active ingredient (89.5% of TK_DDDvet_) could be classed as prophylactic. Sarrazin et al. reported increased treatment frequency at 1, 4 and 9 weeks of age which coincided with birth and associated procedures such as teeth clipping and castration, weaning, and transfer to the finisher stage, respectively [22]. Farmers administer prophylactic treatments at these time points to prevent losses associated with expected disease associated with the stresses of handling, movement and dietary changes [49]. Over half of the study farms administered prophylactic antimicrobial treatments during the first week of life and 88.1% of farms provided antimicrobials in medicated feed at or just after weaning. On 56.7% of farms, medicated feed was provided in the weaner diet. This is fed at the end of the first weaner stage and throughout the second weaner stage and thus covers another transfer between production stages. Therefore, in contrast to Sarrazin et al., where treatments appeared to be administered at strategic times [22], many farms in Ireland administer antimicrobials at, between and after these times. An important reason for this difference is related to the feeding infrastructure. On most farms, a single feed bin supplies one or more houses containing pigs of different ages. If medicated feed is to be provided to the younger pigs just after transfer, the older pigs must be medicated as well. With regard to the weaner and finisher diets, this has important implications for the overall amounts of antimicrobial consumed since the older and heavier the pig, the more it eats. Similar structural issues were reported on Belgian farms using medicated feed [50] and demonstrate the importance of improving antimicrobial delivery systems in efforts to reduce AMU [51].

Quantitative comparisons between studies and national reports are hampered by the use of different indicators of AMU. Use of the mg/PCU indicator, developed by ESVAC [31], allowed a comparison of AMU between the sample population in this study and the 2016 national reports of Sweden, Netherlands, Denmark, France and the UK to be made. Antimicrobial consumption for the combined population of Irish farms was the second highest of the six countries compared. Irish AMU was 3.2 and 2.5 times higher than Denmark and the Netherlands, respectively, when measured in their national indicators. The TI200 indicator allowed a comparison of use at farm level between the study farms and the study by Sarrazin and colleagues [22]. Using purchase data for 1 year, the median TI200 for 180 farrow-to-finish farms from nine countries in 2015 was 7.1. For the Irish farms in 2016, the median TI200 was 15.3. This comparison should be made with caution. The weaner stage on the Irish study farms was generally longer (median = 9 weeks, data not shown) than the European farms (median = 6.5 weeks) [22]. Since most of the AMU is administered in the weaner diet fed to older weaner pigs, the weight at treatment used to calculate the TI_weaner_ is underestimated. This in turn means that the TI200 is overestimated. Also, the farrow-to-finish system accounts for almost all pig production in Ireland [26], and in this regard, the study is representative of the whole industry. In other countries, where specialised weaner and finisher farms are more prominent [26] measuring AMU on farrow-to-finish farms may not be representative. Some studies have found that AMU is higher in finisher pigs on specialised finisher farms than on farrow-finish farms and suggest the stress of transport and mixing pigs from different sources as a possible explanation [12, 19, 52]. Nevertheless, these results show that AMU in Irish pig production compared unfavourably to some of its European peers in 2016. It should be noted, however, that comparable data from many other countries is not yet available and that those countries with AMU data are, in general, further into their respective ‘action plans’ against antimicrobial resistance than Ireland.

These findings highlight the need to reduce AMU in Irish pig production. New EU regulations governing antimicrobial use and medicated feed, which come into effect in 2022, will prevent prophylactic AMU and restrict metaphylactic group treatments as well as the use of CIAs [28, 53]. This will require a significant shift in behavioural and management practices related to AMU on Irish pig farms, particularly with regard to routine prophylactic use in feed medication. A pilot study carried out on an Irish farm suggests this can be achieved without impacting welfare or performance [54, 55] and as an example, if AMU in medicated feed in the weaner, finisher and sow diets were removed from the current study, total AMU for the sample population would reduce to 43.1 mg/PCU which is just below the levels for Denmark and the Netherlands. It should be noted that several farms in the study were already at or below this level of AMU (see Fig. 2); six farms (9% of sample) did not use any medicated feed and seven (10.4% of sample) did not use any category B antimicrobials. The risk factors for AMU on Irish pig farms have not been studied. However, recent work has identified respiratory disease as a significant problem on Irish pig farms [56] and this may explain the high use of antimicrobials in the weaner diets fed to 2nd stage weaner pigs. Also, internal biosecurity on Irish pig farms is lower than in other European countries [57]. Improved biosecurity was shown to aid in reducing AMU on Belgian pig farms [58] and a recent initiative launched by Animal Health Ireland has made biosecurity audits available to all Irish pig farms.^2^ Other initiatives such as measuring AMU, increased education and the promotion of responsible use guidelines, which are all part of iNAP [3], have had success in reducing AMU in pig production in other countries [59]. The recent launch of the ‘national AMU database for pigs’ by the Department of Agriculture Food and the Marine has fulfilled a key priority of iNAP and will allow for the monitoring of AMU in Irish pig production and for the assessment of the impact of AMU reduction strategies [60].

The main limitation of this study is that the data was collected from a convenience sample of farms and thus may not be representative of the entire population. The farms used in this study had originally volunteered to take part in a survey investigating biosecurity and management practices and could represent farms with better practices than the rest of the population which could have introduced a selection bias. Nevertheless, the study population represented around 35% of the national herd and therefore a significant proportion of the industry. Collecting AMU data in the field presents challenges. Records are not always kept [20, 22] and antimicrobial preparations are often used in different age groups without records of which pigs they were allocated to [22] and, therefore, assumptions and imputations had to be made in some instances. There were similar challenges in this study. Where records were incomplete, estimations of AMU were given by the farmer and could be subject to recall bias and intervention bias (where the farmer deliberately under reports AMU). Since the majority of AMU was via medicated feed and is an important part of farm management, the risk of recall bias is considered to be low for this route of administration. Overall, approximately 96% (94% of TK_DDDvet_) of the antimicrobials used could be accurately assigned to the correct age group.

## Conclusions

Antimicrobial use on Irish pig farms is characterised by a high proportion of prophylactic use, primarily delivered using medicated feed and mainly administered to pigs in the post weaning production stages. These patterns of use are similar to those reported in other European studies although the amounts used are higher than some countries such as Sweden, Denmark and the Netherlands. This study confirms that pig production is an important consumer of veterinary antimicrobials, accounting approximately for 40% of AMU in Ireland in 2016; and reinforces the need to reduce AMU. The identification of the pattern of use with the highest impact on consumption, the provision of medicated feed to weaner pigs, suggest that efforts to understand the reasons for this and promote better health among weaner pigs will be of benefit.

## Supplementary information

**Additional file 1: Supplementary Methods**.

**Additional file 2: Supplementary Table 1.** Breakdown of antimicrobial use by stage of production. **Supplementary Figure 1.** Numbers of antimicrobials used in medicated feed in diets for growing pigs on 67 Irish pig farms during 2016. **Supplementary Table 2.** Prophylactic AMU in oral remedies other than premix and injectable preparations.

## Data Availability

The datasets used and/or analysed during the current study are available from the corresponding author on reasonable request.

## Abbreviations

AMU: Antimicrobial use
CIA: Critically important antimicrobial
DAPD: Proportion of animal population in treatment per day
DDD: Defined daily dose
DDD_vet_: Defined daily dose (as defined by the EMA)
DDDA_NAT_: Defined daily dose animal (to define national AMU in the Netherlands)
EMA: European Medicines Agency
ePM: E-Profit Monitor
ESVAC: European Surveillance of Veterinary Antimicrobial Consumption
HP CIA: Highest priority critically important antimicrobial
iNAP: Ireland’s National Action Plan against Antimicrobial Resistance
PCU: Population correction unit
TI: Treatment incidence
TK: Treatable kilograms
UK: United Kingdom
WHO: World Health Organisation

## Footnotes

1 https://www.teagasc.ie/animals/pigs/

2 http://animalhealthireland.ie/?page_id=18234
