# Supplementary material for: Quantification, description and international comparison of antimicrobial use on Irish pig farms
Source: Porcine Health Manag. 2020 Oct 12;6:30. doi: 10.1186/s40813-020-00166-y (PMC7549222; doi:10.1186/s40813-020-00166-y)
Supplement: Supplementary file 1 — Additional file 1: Supplementary Methods. [file 40813_2020_166_MOESM1_ESM.docx]

**Supplementary Methods**

**Calculation of indicators of antimicrobial use**

**mg/PCU**

The mg/PCU uses the weight of active ingredient as the numerator. The population correction unit (PCU) is used as the denominator. The PCU assigns a standardised weight to each species and to sub-categories where applicable [1]. In pigs, standard weights of 25 kg, 65 kg and 240 kg are assigned to weaner pigs, fattening (finisher) pigs and sows respectively. It is specifically calculated using the numbers of breeding animals and numbers of animals sent to slaughter or exported during the period at risk. The number of breeding animals present on the farm, the numbers of weaners sold and the numbers of finishers sent to slaughter were extracted from the Teagasc e-Profit Monitor (ePM) database. Farms that did not supply performance data to the ePM (n = 8) provided the population data directly.

**Treatment incidence**

The calculation of treatment incidence for each age category required repartitioning of each antimicrobial product to the age groups in which it was used. Since medicated feeds are stage specific, all antimicrobials used in medicated feed could be allocated to the intended age category. However, for the other routes of administration it was not possible in every case to determine the amount used in each age category that the product was prescribed for. In such instances, the product was allocated to the relevant age categories in proportion to the weight of biomass of the given age category in accordance with the method used by Sarrazin *et al*. [2].

The kg of animal at risk in each age category was calculated by multiplying the number of animals at risk by assigned weights as proposed by the European Surveillance of Veterinary Anitmicrobial Consumption (ESVAC) project. The assigned weights for piglets, weaners and finishers and sows are 4 kg, 12 kg, 50 kg and 220 kg, respectively [3]. These assigned weights were used in the study by Sarrazin *et* al. [2]. The numbers of animals in each age category were extracted from the ePM database or provided by the farmer directly. The period at risk for each age category was determined by the length of stay which was extracted from the ePM or provided by the farmer directly.

**DAPD**

The DAPD is the indicator used to report antimicrobial use at national level in Denmark. It is defined as the ‘proportion of population in treatment per day’ [4]. DANMAP (Danish Integrated Antimicrobial Resistance Monitoring and Research Programme) assigns a ‘Defined Animal Daily Dose’ (DADD) for each antimicrobial per route of administration [5] and calculates the denominator using production data for the each of the sow (plus piglets), weaner and finisher production categories [5]. The DAPD was calculated for the sample farms by applying the DADD system to the antimicrobial use data and calculating the denominator using estimates of the numbers of animals produced, the period and the average weights in each production category as described by Jensen *et al.* [6].

**DDDA_NAT_**

The DDDA_NAT_ (Defined Daily Dose Animal) is the indicator used to report antimicrobial use at national level in the Netherlands [7]. A ‘Defined Daily Dose Animal’ (DDDA) The DDDA_NAT_ was calculated for the combined sample population as follows. The treatable kilograms were calculated at product level using the DDDA values provided by the Netherlands Veterinary Medicines Institue (SDa) [8]. If an antimicrobial product used in Ireland was not marketed in the Netherlands, then the DDDA of the closest equivalent product with the same active ingredient(s) was used. The denominator was determined by estimating the numbers of animals present in each category (piglets < 20 kg, , fattening pigs, other pigs and sows) and multiplying by the assigned weights (10 kg, 70.2 kg and 70 kg, 220 kg, respectively) [9].

**References**

1. European Medicines Agency. Trends in the sales of veterinary antimicrobial agents in nine European countries. (2011) Available at <https://www.ema.europa.eu/documents/report/trends-sales-veterinary-antimicrobial-agents-nine-european-countries_en.pdf> (accessed on November 19, 2019)
2. Sarrazin S, Joosten P, Gompel LV, Luiken RECE, Mevius DJ, Wagenaar JA, et al. Quantitative and qualitative analysis of antimicrobial usage patterns in 180 selected farrow-to-finish pig farms from nine European countries based on single batch and purchase data. The Journal of antimicrobial chemotherapy. (2019) 74(3):807–16. [doi: 10.1093/jac/dky503](https://app.readcube.com/)
3. European Medicine Agency. Revised ESVAC Reflection Paper on Collecting Data on Consumption of Antimicrobial Agents per Animal Species, on Technical Units of Measurement and Indicators for Reporting Consumption of Antimicrobial Agents in Animals. (2013) EMA/286416/2012-Rev.1. Available at: <https://www.ema.europa.eu/documents/scientific-guideline/revised-european-surveillance-veterinary-antimicrobial-consumption-esvac-reflection-paper-collecting_en.pdf> (accessed on August 2, 2019)
4. DANMAP (Danish Integrated Antimicrobial Resistance Monitoring and Research Programme). DANMAP 2012. Use of antimicrobial agents and occurrence of antimicrobial resistance in bacteria from food animals, food and humans in Denmark. (2013) ISSN 1600-2032. Available at: <https://www.danmap.org/-/media/arkiv/projekt-sites/danmap/danmap-reports/danmap-2012/danmap_2012.pdf?la=en> (accessed on March 3, 2020)
5. DANMAP (Danish Integrated Antimicrobial Resistance Monitoring and Research Programme). DADD description. DANMAP 2018. Use of antimicrobial agents and occurrence of antimicrobial resistance in bacteria from food animals, food and humans in Denmark. (2019) Available at: <https://www.danmap.org/-/media/arkiv/projekt-sites/danmap/danmap-reports/danmap-2018/dadd-beskrivelse_250919.pdf?la=en> (accessed on December 2, 2019)
6. Jensen VF. The question of the denominator: Estimating the live animal population. In: Proceedings of Quantification, Benchmarking and Stewardship of Veterinary Antimicrobial Usage: First International Conference ;27-28 February 2018; Ghent, Belgium. Available at: <https://aacting.org/first-aacting-conference/> (accessed on December 2, 2019)
7. The Netherlands Veterinary Medicines Institute, 2019. Usage of Antibiotics in Agricultural Livestock in the Netherlands in 2018. Trends and benchmarking of livestock farms and veterinarians. <https://cdn.i-pulse.nl/autoriteitdiergeneesmiddelen/userfiles/Publications/2018-def-rapport1.pdf> (accessed April 2, 2020)
8. The Netherlands Veterinary Institute. DG standard. (2019) Available at: https://cdn.i-pulse.nl/autoriteitdiergeneesmiddelen/userfiles/doseringstabel/dg-standaard-30jul2019-tbv-website.pdf (accessed August 2, 2019)
9. The Netherlands Veterinary Institute. Standard operating procedure. Calculation of the DDDA for antimicrobials by the SDa for the cattle, veal, pig, broiler, turkey and rabbit farming sectors. (2020) Available at: <http://cdn.i-pulse.nl/autoriteitdiergeneesmiddelen/userfiles/overige%20rapporten/sop-rekensystematiek-website-03032020.pdf> (accessed April 2, 2020)
