# Supplementary material for: Quantification, description and international comparison of antimicrobial use on Irish pig farms
Source: Porcine Health Manag. 2020 Oct 12;6:30. doi: 10.1186/s40813-020-00166-y (PMC7549222; doi:10.1186/s40813-020-00166-y)
Supplement: Supplementary file 2 — Additional file 2: Supplementary Table 1. Breakdown of antimicrobial use by stage of production. Supplementary Figure 1. Numbers of antimicrobials used in medicated feed in diets for growing pigs on 67 Irish pig farms during 2016. Supplementary Table 2. Prophylactic AMU in oral remedies other than premix and injectable preparations. [file 40813_2020_166_MOESM2_ESM.docx]

**Supplementary Table 1. Breakdown of antimicrobial use by stage of production**

|  | **piglet** | **weaner** | **finisher** | **sow** |
| --- | --- | --- | --- | --- |
| **weight of active ingredient** | 0.9% | 69.7% | 25.4% | 4.1% |
| **treatable kg (DDDvet)** | 2.7% | 63.2% | 30.6% | 3.5% |


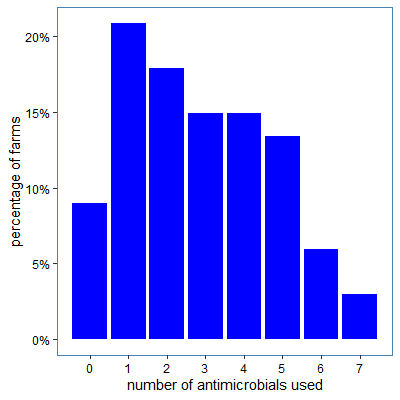


**Supplementary Figure 1.** Numbers of antimicrobials used in medicated feed in diets for growing pigs on 67 Irish pig farms during 2016.

|  | water | top dressing | injectable | all routes |
| --- | --- | --- | --- | --- |
| weight of active ingredient | 78.8% | 39.6% | 11.7% | 95.6% |
| treatable kg (DDDvet) | 76.4% | 38.0% | 23.2% | 89.5% |

**Supplementary Table 2. Prophylactic AMU in oral remedies other than premix and injectable preparations.** The percentages refer to the percentage of AMU in that route category accounted for by prophylaxis.
